# Unraveling Catechol-O-Methyltransferase rs4680 SNP’s Role in Patients’ Response to Tramadol and Its Adverse Effects: A Pharmacogenetics Insight into Postoperative Pain Management

**DOI:** 10.3390/jcm13010249

**Published:** 2023-12-31

**Authors:** Ammara Khan, Akbar Waheed, Tayyaba Afsar, Ali Abusharha, Huma Shafique, Suhail Razak

**Affiliations:** 1 Department of Pharmacology and Therapeutics, Nawaz Sharif Medical College, Gujrat 50700, Pakistan; drammara19@gmail.com; 2 Department of Pharmacology and Therapeutics, Islamic International Medical College, Riphah University, Islamabad 46000, Pakistan; 3 Department of Community Health Sciences, College of Applied Medical Sciences, King Saud University, Riyadh 11495, Saudi Arabia; 4 Department of Optometry, College of Applied Medical Sciences, King Saud University, Riyadh 11495, Saudi Arabia; 5 Institute of Cellular Medicine, Newcastle University Medical School, Newcastle University, Newcastle upon Tyne NE1 7RU, UK

**Keywords:** catechol O-methyltransferase (COMT), tramadol pain, postoperative, adverse drug reaction (ADR), pharmacogenetics

## Abstract

Effective postoperative pain management is essential for patient well-being and an efficient healthcare system. Variations in the Catechol O-Methyltransferase (COMT) gene, specifically rs4680, play a crucial role in pain perception and opioid response. This study seeks to elucidate the impact of rs4680 polymorphism on tramadol efficacy and adverse reactions in post-surgical patients. We performed an uncontrolled cohort pharmacogenetics study in which participants underwent postoperative tramadol administration. The frequencies of rs4680 alleles were determined and the association between rs4680 genotypes and the efficacy of tramadol analgesic as pain relief, measured by the Numeric Rating Scale (NRS), was analyzed. Secondary outcomes included tramadol-induced sedation levels, opioid-induced nausea and vomiting, and other adverse effects of tramadol. Data analysis, using IBM SPSS Statistics 23, focused on pain and side effect differences across genotypes, with statistical significance set to *p* ≤ 0.05. The COMT (rs4680) genotype distribution exhibited a ‘G’ allele frequency of 41.5% and an ‘A’ allele frequency of 58.5%, with the AA genotype present in 44% of individuals, adhering to the Hardy–Weinberg equilibrium (*p* = 0.788). Patients with the AA genotype reported lower pain scores post-tramadol administration across all times examined (*p* < 0.001), but also experienced statistically significant (*p* < 0.001) higher incidences of tramadol-induced nausea, vomiting, and sedation. However, GG genotype individuals experienced poor pain relief from tramadol, requiring more supplemental analgesia. These significant findings underscore the critical role of COMT rs4680 polymorphism in response to tramadol and the necessity of a personalized approach to postoperative pain management.

## 1. Introduction

Acute postoperative pain is described as acute pain in surgical patients stemming from multitude factors involving the activation of nociceptors, the inflammatory response, the activation of the sympathetic nervous system, and processes related to tissue repair and regeneration [1,2]. The World Health Organization and the International Association for the Study of Pain have acknowledged the treatment of postoperative pain as a fundamental human right that will not only enhance patient recovery and well-being but also lessen the economic burden on already constrained healthcare systems in developing countries [3]. However, despite strong recommendations, adequate post-operative pain control is still unachieved in almost 70 to 80% of surgical patients, not only in developing countries but all around the world [4,5].

Advancements in opioid-free/sparing techniques, such as neuroaxial blocks and regional anesthesia, are reshaping postoperative pain management, aligning well with Enhanced Recovery After Surgery (ERAS) protocols. However, there remains a significant portion of postoperative care where traditional opioid-based analgesics like tramadol are utilized, given limited resources and techniques. In this context, understanding pharmacogenetics, which plays a crucial role in impacting the efficacy and safety of administered opioid analgesics, including tramadol, is imperative [6].

Tramadol is a multifaceted analgesic that is not only an agonist at mu-opioid receptors (MORs), but also elevates synaptic levels of neurotransmitters, namely serotonin (5-HT) and norepinephrine (NE), by inhibiting their respective transporters—NET and SERT [7]. Both neurotransmitters are metabolized by the enzyme Catechol-O-methyltransferase enzyme (COMT), and encoded by the COMT gene. Genetic polymorphisms within the COMT gene lead to altered enzymatic activity, resulting in varied pain sensitivities among patients with conditions such as fibromyalgia, chronic migraines, and temporomandibular joint dysfunction [8,9]. Interestingly, genetic polymorphisms within the COMT gene are also associated with diverse responses to drugs, including opioids, cisplatin, and methylphenidate [10,11]. The rs4680 single nucleotide polymorphism (SNP) is noteworthy as it has a global minor allele frequency of 37%. This SNP causes the nucleotide guanine (G) at position 472 to change to adenine (A), which translates into the amino acid valine (Val), a replacement for methionine (Met). This results in the production of a thermolablile COMT enzyme with decreased activity [12]. Research has indicated that AA genotyped individuals have an upregulation of MORs, leading to enhanced analgesia and side effects from opioids [13]. They need a smaller dose of opioids to manage cancer-related [8] and post-operative pain with fewer adverse effects [14]. This effect of polymorphism is not only limited to homozygous individuals but also extends to heterozygous (GA) mutations, as a study documented that these patients consume significantly less morphine in the post-anesthetic recovery phase and up to 48 h post-operation compared to those with homozygous (GG) variants [15,16]. Additionally, these patients have recorded lower nausea and sedation scores across all monitored postoperative time points [15,16]. However, the association of the COMT rs4680 SNP with the enhanced efficacy of opioids is not always consistent, as a meta-anaylsis document revealed no significant association between COMT rs4680 SNP and opioid efficacy in post-operative settings [17].

Given the above conflicting data regarding the influence of rs4680 SNP on opioid efficacy and adverse reactions, and the limited data for tramadol, further investigation into this polymorphism’s role in pain management is important. Our study aims to examine the prevalence and impact of rs4680 variations in the COMT gene on tramadol’s efficacy and adverse reactions to postoperative pain management. This research seeks to enhance our understanding of the genetic factors in pain management, potentially leading to more personalized and effective postoperative pain strategies, thereby improving patient care and outcomes.

## 2. Methods

This study was a joint initiative between the Department of Pharmacology and Therapeutics at Islamic International Medical College, Riphah International University, and the Departments of Surgery at Nawaz Sharif Medical College, University of Gujrat. The experimental protocols underwent thorough examination and received approval from the ethical review committee (ERC) of Riphah International University (Riphah/IRC/20/103), and the Institutional Review Board (IRB) of Nawaz Sharif Medical College to ensure ethical norms and university guidelines were met, the protection of participant privacy, and the reduction of participant risk.

### 2.1. Study Design

This was an Uncontrolled Cohort Pharmacogenetics Study that allowed us to explore the relationship between individual genetic variations and differential responses to a specific pharmacological intervention, thus providing insights into personalized medicine’s potential [18]. With an MAF for the target SNP of 0.30, a significance level set at 0.05%, and a study power of 80%, the required sample size was determined to be 100 people, who were recruited randomly [19,20]. Informed consent was taken from each study participant before including them in the study.

### 2.2. The Inclusion Criteria and Exclusion Criteria

The study included post-operative patients of both genders above 18 years of age, undergoing uncomplicated open appendectomy and cholecystectomy under general anesthesia. Open appendectomies, though sometimes emergent, were managed within a timeframe that allowed full adherence to study protocols without compromising patient safety. Eligibility also required the ability and willingness of the participants to use the numeric rating scale (NRS) to report post-operative pain intensities

Exclusion criteria were equally stringent, with patients showing history of liver or kidney diseases, drug abuse, seizures, psychiatric disorders, respiratory issues, chronic alcohol consumption, and regular opioid administration being excluded. Patients with potential allergic reactions to tramadol, inability to provide informed consent, and who required unscheduled emergency surgery were also excluded to maintain the homogeneity of the study group.

### 2.3. Sampling

Before the administration of anesthesia, a peripheral venous blood sample was collected into an EDTA-coated tube for genetic analysis. All study participants were premedicated with midazolam (0.1 mg/kg), along with receiving standard hospital care. Anesthesia was standardized in all patients. Induction was performed with propofol (1–3 mg/kg) and atracurium (0.5 mg/kg), followed by maintenance with sevoflurane or isoflurane, combined with a mixture of oxygen and air. Intraoperative opioid-free analgesia was achieved using ketamine (0.3 to 0.5 mg/kg), paracetamol (1 g), and a TransAbdominal Plane (TAP) block with short acting local anesthetics for effective pain control, particularly suited to the major abdominal surgeries in our study. This multimodal approach aimed to optimize pain management during surgery while minimizing potential complications and confounding factors when judging post-operative tramadol efficacy. Tramadol, 50 mg in 50 mL of 0.9% sodium chloride (NaCl) every six hours, was administered to all patients for post-operative analgesia. We carefully evaluated all study patients post-operatively for anesthesia-related complications and tramadol induced side effects. Rescue analgesia in the form of paracetamol 1 gm IV was administered to patients reporting pain above score 5–6 despite tramadol administration. In the case of tramadol-induced emesis, intravenous dimenhydrinate was utilized.

### 2.4. Dependent Variable Assessment

We designed a patient form to collect the data, which included information on the demographics, medical history, drug history, and clinical parameters of each participant. A pilot test was conducted with a select group of participants to validate the form, ensuring it was comprehensive and user-friendly. This ensured we had detailed and relevant information for our analysis. The study participants were also given instructions and the post-operative recording methods were explained to them before the surgical procedure. The study participants were carefully evaluated at 5 pre-determined times: at the 1st, 3rd, 6th, 12th and 24th hour post-surgery [21]. To assess the analgesic efficacy of tramadol, pain scores were assessed at rest and during movement using a validated numeric rating scale (NRS) [22]. Another validated and sensitive tool, a simplified version of the Pasero Opioid-induced Sedation Scale (POSS), was employed for assessing tramadol induced sedation [23]. If the patient showed no reaction to a physical stimulus due to excessive sedation, the protocol was to cease tramadol treatment and administer naloxone. A simplified ordinal scale was chosen for opioid-induced nausea and vomiting (OINV) assessment. The scale consisted of level 0 (absence of vomiting or nausea sensations), level 1 (presence of nausea sensations without episodes of vomiting), and level 3 (presence of both nausea sensations and episodes of vomiting) [24,25]. Other potential side effects of tramadol, including, dizziness, headaches, sweating, and dry mouth, were also evaluated. These recordings were entered in the patient form by the clinical team.

### 2.5. Genetic Analysis

We extracted DNA from the stored blood samples utilizing the phenol–chloroform method [26]. The separated DNA fragments were later analyzed with Gel electrophoresis. We prepared the gel by mixing agarose with TAE buffer and adding Ethidium Bromide for DNA visibility. After solidification, we added DNA samples to the loading dye and conducted electrophoresis at 70 volts for 60 min, visualizing the results using a UV Trans-Illuminator. Post electrophoresis, the 260/280 nm absorbance ratio of extracted DNA, to assess its purity, was determined using a Thermo Scientific Multi Skan Go Instrument (Waltham, MA, USA). Once extracted, the DNA region of interest was amplified via PCR utilizing site specific primers. The forward primers for our selected SNP rs4680 had TM of 52 °C and sequence of “GAAGGGTGGAAAAGATAGGG”, whereas the reverse primer had TM of 50 °C and sequence of “CTTTAGGGTTCTGGGATGAC”. The PCR reaction was carried out in a Galaxy XP Thermal Cycler. In the PCR process, a master mix containing DNA polymerase, buffer, dNTPs, Mg2+ ions, and other components was used for consistent and accurate amplification. After the PCR amplification, the resulting PCR products were subjected to gel electrophoresis to confirm specific binding and product size. In the case of non-specific amplification, the PCR reaction was fine-tuned including gradient PCR approach and the addition of 1% DMSO in the master mix. Subsequently, Sanger sequencing was employed to analyze the purified PCR products using Applied Biosystems’ (Waltham, MA, USA) 3730xl DNA Analyzer [27]. Sequencing reactions were set up using the purified PCR product, a target specific primer, DNA polymerase, and a mix of regular and fluorescently labeled dideoxynucleotides (ddNTPs). Post-reaction, capillary electrophoresis was performed to segregate the DNA fragments by size, and the resultant chromatogram was analyzed by Applied Biosystems’ Sequence Analysis BioEdit Software. A manual review of the sequencing data was conducted to ensure accuracy, particularly in regions around the SNP. For SNP identification and validation, we utilized bioinformatics tools such as NCBI’s BLAST and SeqMan (DNASTAR), comparing our sequences against reference genomes to identify and confirm the presence of SNPs. Throughout the procedure, strict measures were taken to avoid contamination, and all protocols were carried out with adherence to safety guidelines.

### 2.6. Statistical Analysis

In our study, we used IBM SPSS Statistics version 23 for statistical analysis, focusing on continuous variables like allele frequencies and pain scores, and categorical variables including genotypic classifications and clinical outcomes. We employed Chi-square and exact tests to assess Hardy–Weinberg Equilibrium, with a *p*-value over 0.05 indicating alignment. Z-tests were used to compare allele frequencies with global datasets, considering *p*-values below 0.05 as significant. For continuous data, ANOVA and Levene’s Test were used, with post-hoc analyses (Tukey or Games–Howell) based on data distribution. Categorical outcomes like OINV, sedation scores, and other side effects were analyzed using Pearson and Chi-Square tests or Fisher’s Exact Test for small cell counts. A *p*-value of less than 0.05 was taken as significant throughout the study.

## 3. Results

We recruited 105 patients as per our inclusion criteria. Five of them were unable to complete the study due to an error in sampling time. The demographic data set, as given in Table 1, provides insights into a population characterized by a predominance of females with mean age of almost 35 years, and indicate a health sample.

### 3.1. Allele Frequency of COMT Rs4680

In evaluating the distribution of the COMT (rs4680) genotype within our sample population, the frequency of the presence of the ‘G’ allele was 41.5% and that of the ‘A’ allele was 58.5%. The homozygous AA genotype was observed in 44% of the study participants, the AG genotype in 29%, and the GG in 27% of the study participants. A high *p*-value of 0.788 suggests that the observed genotype frequencies in the study sample do not significantly deviate from the expected frequencies under the Hardy–Weinberg equilibrium (Table 2).

### 3.2. Comparing rs4680 Allele Frequencies with Global Data

**Figure 1:**
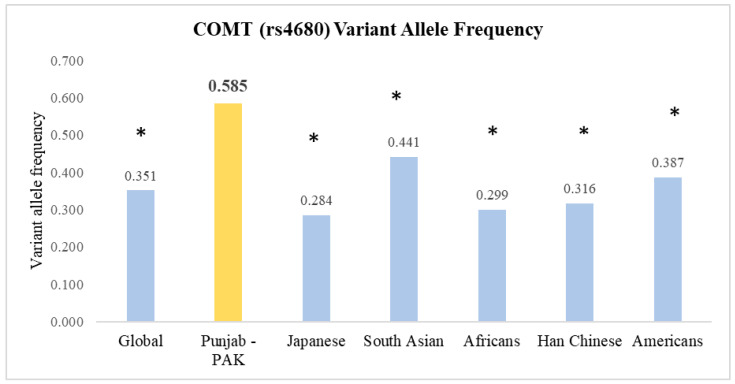
Comparison of the frequencies of rs4680 allele variants between the Punjab, Pakistan Population and other global populations. * *p* < 0.05, when compared to our study population.

The frequency of the ‘A’ variant allele in our population was statistically significantly (*p* < 0.05) different from the other set of populations, as per the 1000 Genomes Project Phase 3 [28]. When compared with the global frequency of 0.351, the discrepancy was significant (Figure 1). Similarly, comparisons with other populations, like the Japanese (0.284), South Asians (0.441), Africans (0.299), Han Chinese (0.316), and Americans (0.387), showed significant differences. A bar chart was constructed to present the allele frequencies across different populations graphically and associated *p*-values from the z-tests for proportions are displayed above each bar, providing a clear visual indication of statistical significance (Figure 1).

### 3.3. Post-Operative Pain Scores at Rest and on Raising Head by COMT (rs4680) Genotype

The effect of rs4680 on tramadol efficacy for postoperative pain was assessed by comparing pain scores both at rest and upon movement at various time points among three genotypes (Table 3). In all the observed time frames (1, 3, 6, 12, and 24 h post-operation), the AA genotype group consistently reported lower pain scores both at rest and during movement following tramadol administration. As shown in Table 3, pain scores recorded at rest, 1 h post-operation, in the AA genotype group (5.704 ± 1.090) were statistically significantly (*p* < 0.001) lower than that in GG genotype group (8.59 ± 0.628). The AG group demonstrated intermediate pain scores of 6.63 ± 1.27, which was statistically different from both the GG and AA genotype groups (*p* < 0.001). A similar observation was made for pain scores upon movement. This trend, in which study participants with the AA genotype experienced lower pain levels, both at rest and upon movement, than those in the GG group was observed during every recording. Each of these observed differences was statistically significant (*p* < 0.001).

The supplemental analgesia requirement also demonstrated the same effect, with the GG group demanding more rescue analgesia, followed by AG genotyped patients. The AA group utilized minimal rescue analgesia, supporting their lower pain scores across all the time points examined.

### 3.4. Incidence of Nausea and Vomiting following Tramadol Administration Based on COMT (rs4680) Genotype at Various Post-Operative Time Points

Our data indicate that participants with the AA genotype of COMT (rs4680) polymorphism had a statistically significant increase in their susceptibility to tramadol induced nausea and vomiting (Table 4).

At one hour after surgery, half (50%) of the patients with the AA genotype experienced nausea, statistically significantly (*p* < 0.001) more than those with the GG (7.4%) genotype. Vomiting was also observed more often in the AA genotype (20.5%) and AG (3.4%) genotype groups. Whereas no report of vomiting was seen in the GG group. At the third hour of recording, the incidence of nausea declined to almost 32% for the AA group; however, it was still significantly (*p* < 0.05) higher than the GA (24.1%) and GG (3.7%) groups. Additionally, about 36.4% of the AA genotype group also vomited, significantly higher than both the GA (6.9%) and GG (0%) groups (*p* < 0.05). With the passage of time, a significant reduction in nausea and vomiting was observed across all the groups. However, the AA group still reported the highest incidence of nausea and vomiting, followed by GA genotype group, with a statistically significant *p* value of < 0.01 for AA vs. GA and GG at 6 h of recording. Interestingly, no patient with the GG genotype reported nausea. A similar pattern was observed in the latter recordings.

### 3.5. Sedation Scores in Patients following Tramadol Administration Based on COMT (rs4680) Genotype

The evaluation of tramadol-induced sedation among three genotypes at five post-operative points, also revealed an intriguing trend (Table 5). Just like tramadol-induced vomiting, the AA and GA genotype groups exhibited increased sedation at the 1 h post-operative recording. Whereas a significant number of GG genotype patients were awake (40.7%) as compared to only 17.2% of GA and 0% of AA genotype patients, a statistically significant difference (*p* = 0.014). Similarly, at the 3 h post-operative recording, the majority of AA and AG genotype patients were sedated because of tramadol while all of GG genotyped patients had no tramadol-induced sedation, demonstrating the genotypes’ continued influence on alertness (*p* < 0.001 for all comparisons, except GA vs. GG: *p* = 0.083). These differences in sedation levels attenuated over time; however, a significant fraction of those with the AA genotype who were still sedated by tramadol were aroused easily with verbal commands, underscoring the enduring effect of the genotype on sedation levels.

### 3.6. Incidence of Other Side Effects following Tramadol Administration among COMT (rs4680) Genotypes

Our investigation into the other side effects of tramadol administration, like headaches, dizziness, dry mouth, and sweating, revealed noteworthy differences in their manifestation across different genotypes (Table 6). A recurring adverse effect, dizziness, was predominant among GG genotype participants with a significant 33% of cases occurring 1 h post-operatively, with statistically significant *p*-values between genotypes (GG vs. GA: *p* = 0.034). In contrast, the AA and GA genotype groups were largely resilient, demonstrating minimal occurrences throughout all the recordings.

Headaches were another important side effect, particularly among individuals with the GG genotype. At 3 h post operation, 37% of GG individuals experienced headaches, significantly higher than the GA and AA genotypes.

Sweating and a dry mouth were less frequent but were still notably present, especially in the GG and GA genotype groups, occasionally exhibiting a significance in their *p*-values.

## 4. Discussion

The therapeutic effectiveness of tramadol has been extensively demonstrated in various surgical contexts for post-operative pain, as it holds analgesic effectiveness comparable to morphine but with a significantly lower incidence of opioid-induced side effects, especially respiratory depression [29,30]. However, the efficiency of tramadol is subject to individual genetic variations including rs4680 polymorphism in the COMT enzyme, highlighting the role of pharmacogenetics in the pain management [31]. Given the scarcity of data relevant to different demographics, we conceptualized our study and went on to identify the rs4680 SNP and its impact on tramadol’s efficacy against postoperative pain.

Our data revealed that the frequency of the “A” allele variant was observed in 58.5% of the studies’ population, a significant contrast to 28–40% range seen in our neighboring Asian populations and globally [28]. Regarding the genotypes, a homozygous AA genotype was observed in 44% of the cohort, indicating that almost half of our studied population had altered COMT enzymatic activity, underscoring the relevance of our research objective. The identified genotypic distribution was in accordance with the Hardy–Weinberg equilibrium, suggesting that the observed frequencies of the genotypes in the studied population are representative and are not due to genetic linkage or selection [32].

The enzyme catechol-O-methyltransferase (COMT), central to the metabolism of biogenic amines, has a significant role in pain perception and can impact the pain-relieving effects of tramadol [33]. This pain modulation involves the complex interplay of adrenergic neurotransmitters in peripheral, spinal, and supraspinal areas. For instance, NE, can either enhance pain through β-adrenergic receptors or mitigate it via α2-adrenoceptors by modulating inflammatory responses and beta-endorphin release. Studies highlight NE’s role in spinal and supraspinal pain relief in both animal and human cancer patients, which is influenced by its interaction with α2 and µ-opioid receptors [34,35,36]. Dopamine, another substrate for COMT, activates descending inhibitory pain pathways in the periaqueductal gray (PAG) region [34,35]. Our study contributes to this understanding by correlating the COMT genotype with tramadol’s pharmacodynamics, which is particularly relevant in the context of acute postoperative pain and may extend to chronic pain management.

Our research aligns with previous studies which have associated diminished COMT activity with an upregulation of opioid receptors in rs4680 SNP and consequently led to alterations in the analgesic and side effect profiles of opioids like tramadol [11]. The findings from our study reveal that the AA (Met/Met) genotype study population experienced the least amount of pain both at rest and upon movement, and thus experience enhanced analgesic efficacy with tramadol. Conversely, individuals possessing the GG (Val/Val) genotype consistently experienced elevated levels of pain at rest and upon movement, followed by those with the AG (Val/Met) genotype. Importantly, these variations were statistically significant, particularly when comparing the GG group with the AA group, and the AG group with the AA group. A comparable scenario was presented by Lecia M. Nielsen and colleagues, who inferred that individuals possessing the G allele of the COMT rs4680 polymorphism exhibited a heightened susceptibility to pain in contrast to their A allele counterparts, implying that carriers of the G (Val) allele might require higher doses of analgesic medications, such as morphine, to manage pain effectively [37]. Another work by Anne Tammimäki and colleagues confirmed that decreased COMT activity in rs4680 SNP is associated with the upregulation of opioid receptors, thereby enhancing the analgesic effects of opioids, along with their side effects [8]. In a similar vein, research conducted by Rebecca L Andersen and associates pointed out that individuals harboring the AA (Met/Met) genotype manifested an increased responsiveness to opioids, including tramadol, albeit with an escalation in associated adverse effects [38]. More support for our data comes from the evidence-based review that suggested that the COMT rs4680 polymorphism has a considerable impact on the efficacy of opioid therapy in the context of acute post-operative pain and chronic pain scenarios. More precisely, the COMT rs4680 polymorphism was accorded a grade B recommendation, given that individuals with diminished COMT activity might exhibit an increased number of opioid receptors, which could potentially amplify the analgesic effects and side effect profiles of opioids [39].

Nevertheless, the higher neurotransmitter levels and increased μ-opioid receptors in COMT rs4680 SNP could also heighten the likelihood of patients experiencing side effects from tramadol, including nausea, dizziness, and sedation. This is evident from our study’s results as the ‘A’ allele had a pro-emetic effect and the AA and AG genotype study groups experienced a higher incidence of nausea and vomiting across all post-operative time interval recordings. This might be linked to the diminished breakdown of dopamine by the altered COMT enzyme, which is significant in the emetic response [40]. Furthermore, tramadol’s inhibition of SERT and subsequent activation of 5-HT3 receptors in the gastrointestinal tract (GIT) and chemoreceptor trigger zone (CTZ), along with enhanced activity at upregulated μ-opioid receptors, are key contributors to OINV in this context [24]. Our results are supported by research documenting the heightened incidence of aura with nausea and vomiting in migraine patients with the AA rs4680 SNP genotype [41]. Similarly Laugsand and colleagues assessed the role of rs4680, along with multiple other SNPs across 16 genes, in opioid induced nausea and vomiting among cancer patients, and concluded that patients with the ‘G’ allele experienced less tramadol induced nausea and vomiting [42].

The reduced enzymatic activity of COMT in rs4680 polymorphism also augments the sedative effects of tramadol, as is evident from our study. Based on the findings of our research, individuals possessing the AA genotype exhibited a heightened sensitivity to the sedating properties of tramadol. The activity of COMT is pivotal in modulating dopamine levels in the pre-frontal cortex, where it metabolizes around 60% of dopamine, influencing sleep and cognitive functions [35,43]. Studies have documented that individuals with an AA genotype exhibit increased alpha power in EEG, usually associated with superior sleep quality [44]. Another study found an association between the G (Val) allele and a reduction in fast spindle density during sleep, which is crucial in maintaining sleep quality [45]. This, coupled with upregulated MOR in rs4680 SNP in the face of reduced met –enkephalin levels, further intensifies tramadol’s sedative effects in AA genotyped patients [40].

Our evaluation of tramadol administration reveals distinctive patterns in side effect occurrences across the GG, GA, and AA COMT genotypes (rs4680). Throughout the post-operative periods, our study revealed that the GG genotype patients recurrently experienced a spectrum of adverse effects including a higher incidence of headaches and dizziness. Whereas the GA genotype remains moderately resilient, the AA genotype primarily remains free of side effects.

To summarize, our investigation highlights the detailed impact of rs4680 polymorphism in the COMT gene on the efficacy of tramadol and its adverse reaction profile, resulting in invaluable insights which demand careful consideration when designing therapeutic strategies. Our findings not only bridge the existing knowledge gap within our set of populations but also underscore the importance of pharmacogenomic approaches in optimizing pain management protocols, potentially mitigating adverse drug reactions, and enhancing the quality of patient care.

Despite the insight provided, our study is not without limitations. The single-centered and population-specific nature of our research may limit the generalizability of our findings across diverse populations and ethnic groups, necessitating multi-center and multi-ethnic investigations for broader applicability. Importantly, the exclusion of chronic pain patients, who are at risk of postoperative hyperalgesia, and the lack of investigation into long-term effects of genotype-based dosing are notable limitations. By concentrating solely on rs4680 polymorphism in the COMT gene, we might have overlooked other genetic variations that could play a role in reactions to tramadol, suggesting a need for broader genomic studies that include multiple genes and polymorphisms. Additionally, the study did not account for external variables like lifestyle, dietary habits, and environmental factors which could interact with genetic predispositions and impact drug responses. Lastly, the study’s sample size was relatively modest, and larger-scale studies are crucial to validate our findings and to detect subtle genetic influences with higher precision.

## 5. Conclusions

Our study reveals that the COMT rs4680 SNP significantly influences tramadol’s effectiveness and side effects in postoperative pain management in our study population. Individuals with the AA genotype experienced enhanced pain relief from tramadol; however, this was at the cost of increased tramadol-induced nausea and sedation, while those with the GG genotype showed a lower incidence of nausea but a higher tendency for dizziness and headaches. These findings highlight the potential of pharmacogenetics in personalizing pain management, suggesting the rs4680 SNP as a key player in tailoring analgesic therapy and bridging the pharmacogenetics knowledge gap in given demographics and locations. However, further research with a wider array of genetic markers is necessary to deepen our understanding of individualized pain treatment.

## Figures and Tables

**Table 1:** Demographic data of the study.

Demographic Data		
Parameters	Characteristics	Value
**Gender**	Male Female	37% 63%
**Material Status**	Married Unmarried	75% 25%
**Mean Age (Years)**		34.95 ± 13.47
**Mean Weight (Kg)**		70.77 ± 11.13
**Mean Height (feet)**		5.45 ± 0.28
**Mean Body Mass Index**		25.12 ± 4.15
**Smoking Status**	Yes No	5% 95%
**Mean Serum Creatinine**		0.78 ± 0.20
**Mean Urea**		28.32 ± 7.81
**Previous Medication**	Yes No	2% 98%
**Mean Serum ALT**		38.01 ± 21.32
**Mean Serum AST**		39.03 ± 22.00
**Alcohol consumption**		0%
**ASA Status**	ASA Status I. Healthy II. Mild Systemic Disease III. Sever Systemic Disease	69% 29% 2%
**Duration of Surgery (Minutes)**		30.82 ± 4.70

**Table 2:** Frequency of reference and variants of COMT Rs4680 alleles. p^HWE^ > 0.05.

Gene	SNP	Allele Frequency (%)	Genotype/Haplotype	Observed Frequency (%)	Expected Frequency (%)	95% Confidence Interval	p^HWE^
COMT (G/A) rs4680	G	83 (41.5%)	G/G	27	17	0.186–0.368	0.788
	A	117 (58.5%)	A/G	29	49	0.204–0.389	
			A/A	44	34	0.341–0.543	

**Table 3:** Post-operative pain scores at rest and upon raising head by COMT (rs4680) Genotype. * Mean ± S.D. ** *p* value ANOVA. *** *p* value post hoc Tukey test. 95% CI in brackets.

Post-Operative Time	COMT (rs4680)			*p*-Value **	GG vs. GA ***	GG vs. AA ***	AA vs. GA ***
	GG * (*n* = 27)	GA * (*n* = 29)	AA8 (*n* = 44)				
**Pain Score at Rest**							
**1 h**	8.59 ± 0.628	6.63 ± 1.275	5.704 ± 1.090	<0.001	**<0.001** (0.99, 2.39)	**<0.001** (−3.46, −2.17)	**0.001** (−1.7511, −0.496)
3 h	7.069 ± 1.193	5.59 ± 1.474	4.50 ± 0.999	<0.001	**<0.001** (0.54, 2.09)	**<0.001** (−3.23, −1.81)	**0.001** (−1.89, −0.504)
6 h	5.896 ± 1.046	4.629 ± 1.42	3.863 ± 0.979	<0.001	**<0.001** (0.356, 1.83)	**<0.001** (−2.66, −1.32)	**0.018** (−1.554, −0.236
12 h	5.827 ± 0.848	4.148 ± 1.262	3.091 ± 0.884	<0.001	**<0.001** (0.806, 2.13)	**<0.001** (−3.29, −2.09)	**<0.001** (−1.810, −0.629)
24 h	5.241 ± 1.214	3.778 ± 1.502	2.500 ± 0.44	<0.001	**<0.001** (0.212, 1.87)	**<0.001** (−3.33,−1.82)	**<0.001** (−2.27, −0.795)
**Pain Score at movement (Head Raising)**							
1 h	8.69 ± 0.761	7.11 ± 1.87	6.204 ± 1.25	<0.001	**<0.001** (0.69, 2.15)	**<0.001** (−3.13, −1.79)	**0.003** (−1.69, −0.387)
3 h	7.103 ± 1.52	5.33 ± 1.09	5.159 ± 0.805	<0.001	**<0.001** (1.18, 2.58)	**<0.001** (−2.74, −1.46)	0.798 (−0.848, 0.407)
6 h	6.65 ± 1.11	5.00 ± 1.00	4.59 ± 0.731	<0.001	**<0.001** (0.876, 2.11)	**<0.001** (−2.69, −1.57)	0.076 (−1.19, −0.087)
12 h	6.34 ±0.936	4.74 ± 0.98	3.818 ± 1.04	<0.001	**<0.001** (0.89, 2.19)	**<0.001** (−3.14, −1.96)	**0.001** (−0.087, −0.43)
24 h	5.68 ± 0.806	4.07 ± 0.729	2.863 ± 1.002	<0.001	**<0.001** (0.79, 1.98)	**<0.001** (−3.31, −2.22)	**0.001** (−1.91, −0.845)

**Table 4:** Incidence of nausea and vomiting following tramadol administration based on COMT (rs4680) genotype at various post-operative time points. * Cell values represent the number and percentage of participants within each genotype group experiencing OINV at the respective time point. ** the *p*-values are obtained using Pearson Chi-Square test. Fisher’s exact *p*-value is reported when the expected cell count is less than 5.

Nausea Vomiting 30 Minutes after Tramadol							
	COMT (rs4680)			*p* Value **	GG vs. GA **	GG vs. AA **	AA vs. GA **
	GG (27) *	GA (29) *	AA (44) *				
**1 h post-operatively**							
**No Nausea (*n* = 48)**	25 (92.6%)	10 (34.5.%)	13 (29.5%)	**<0.001**	**<0.001**	**<0.001**	0.152
**Feel Nausea** **(*n* = 42)**	2 (7.4%)	18 (60.1%)	22 (50%)				
**Vomit (*n* = 10)**	0 (0%)	1 (3.4%)	9 (20.5%)				
**3 h post-operatively**							
**No Nausea (*n* = 60)**	26 (96.3%)	20 (69%)	14 (31.8%)	**<0.001**	**0.027**	**<0.001**	**0.03**
**Feel Nausea (*n* = 22)**	1 (3.7%)	7 (24.1%)	14 (31.8%)				
**Vomit (*n* = 18)**	0 (0%)	2 (6.9%)	16 (36.4%)				
**6 h post-operatively**							
**No Nausea (*n* = 68)**	26 (96.3%)	24 (82.8%)	18 (40.9%)	**<0.001**	0.134	**<0.001**	**0.002**
**Feel Nausea (*n* = 21)**	0 (0%)	4 (13.8%)	17 (38.6%)				
**Vomit (*n* = 11)**	1 (3.7%)	1 (3.4%)	9 (20.5%)				
**12 h post-operatively**							
**No Nausea (*n* = 92)**	23 (85.2%)	25 (86.2%)	23 (52.3%)	**0.026**	-	0.062	**0.05**
**Feel Nausea (*n* = 7)**	4 (14.8%)	4 (13.8%)	20 (4%)				
**Vomit (*n* = 1)**	0 (0%)	0 (0%)	1 (2.3)				
**24 h post-operatively**							
**No Nausea (*n* = 93)**	27 (100%)	28 (100%)	38 (91%)	0.062	1.00	0.076	0.232
**Feel Nausea (*n* = 7)**	0 (0%)	1 (0%)	6 (9%)				
**Vomit (*n* = 0)**	0 (0%)	0 (0%)	0 (0%)				

**Table 5:** Sedation scores in patients following tramadol administration based on COMT (rs4680) genotype. * Cell values represent the number and percentage of participants within each genotype group experiencing sedation level category at the respective time point. ** the *p*-values are obtained using Pearson Chi-Square test. Fisher’s exact *p*-value is reported when the expected cell count is less than 5. ^#^ no variation in sedation level/constants.

Sedation Level							
	COMT (rs4680)			*p* Value **	GG vs. GA **	AA vs. GG **	GA vs. AA **
	GG (27) *	GA (29) *	AA (44) *				
1 h post-operatively							
Awake (*n* = 16)	13 (48.1%)	2 (6.9%)	1 (2.3%)	**<0.001**	**0.001**	**<0.001**	0.078
Easily Awakened by Verbal Command (*n* = 57)	13 (48.1%)	21 (72.4%)	23 (52.3%)				
Difficulty in staying awake (*n* = 27)	1 (3.7%)	6 (20.7%)	20 (40.4%)				
No awakening (*n* = 0)	0 (0%)	0 (0%)	0 (0%)				
3 h post-operatively							
Awake (*n* = 67)	27 (100%)	26 (89.6%)	14 (31.8%)	**<0.001**	0.083	**<0.001**	**0.001**
Easily Awakened by Verbal Command (*n* = 33)	0 (0%)	3 (10.4%)	30 (68.2%)				
Difficulty in staying awake (*n* = 0)	0 (0%)	0 (0%)	0 (0%)				
No awakening (*n* = 0)	0 (0%)	0 (0%)	0 (0%)				
6 h post-operatively							
Awake (*n* = 87)	27 (100%)	29 (99%)	32 (72.7%)	**<0.001**	0.330	**0.002**	**0.01**
Easily Awakened by Verbal Command (*n* = 13)	0 (0)	1 (1%)	12 (27.3%)				
12 h post-operatively							
Awake (*n* = 88)	27 (100%)	28 (96.6%)	33 (75%)	**0.002**	0.330	**0.005**	**0.02**
Easily Awakened by Verbal Command (*n* = 12)	0 (0%)	1 (3.4%)	11 (25%)				
24 h post-operatively							
Awake (*n* = 99)	29 (100%)	27 (100%)	43 (99%)	0.273	Not possible ^#^	0.522	0.224
Easily Awakened by Verbal Command (*n* = l)	0 (0%)	0 (0%)	1 (1%)				

**Table 6:** Incidence of other side effects following tramadol administration among COMT (rs4680) genotypes. * Cell values represent the number and percentage of participants within each genotype group experiencing adverse effects at the respective time point. ** the *p*-values are obtained using Pearson Chi-Square test. Fisher’s exact *p*-value is reported when the expected cell count is less than 5.

Other Side Effect of Tramadol Administration							
	COMT (rs4680)			*p* Value **	GG vs. GA **	AA vs. GG **	AA vs. GA **
	GG (*n* = 27) *	GA (*n* = 29) *	AA (*n* = 44) *				
1 h post-operatively							
No side effects (*n* = 70)	12 (44.4%)	22 (75.8%)	36 (81.8%)	0.008	0.034	0.003	0.326
Dizziness (*n* = 16)	9 (33.3%)	2 (6.9%)	5 (11.4%)				
Headache (*n* = 11)	5 (18.5%)	4 (13.8%)	2 (4.5%)				
Sweating (*n* = 2)	1 (3.7%)	1 (3.4%)	0 (0%)				
Dry Mouth (*n* = 1)	0 (0%)	0 (0%)	1 (2.3%)				
3 h post-operatively							
No side effects (*n* = 78)	15 (55.5%)	24 (82.7%)	39 (88.6%)	0.001	0.02	0.001	0.424
Dizziness (*n* = 2)	0 (0%)	0 (0%)	2 (4.5%)				
Headache (*n* = 14)	10 (37%)	2 (10.3%)	2 (4.5%)				
Sweating (*n* = 2)	1 (3.7%)	1 (3.4%)	0 (0%)				
Dry Mouth (*n* = 4)	1 (3.7%)	2 (10.3%)	1 (2.3%)				
6 h post-operatively							
No side effects (*n* = 81)	17 (63%)	24 (82.7%)	40 (90.9%)	0.02	0.439	0.003	0.248
Dizziness (*n* = 3)	1 (3.7%)	0 (0%)	2 (4.5%)				
Headache (*n* = 11)	7 (25.9%)	3 (10.3%)	1 (2.3%)				
Sweating (*n* = 2)	1 (3.7%)	1 (3.4%)	0 (0%)				
Dry Mouth (*n* = 3)	1 (3.7%)	1 (3.4%)	1 (2.3%)				
12 h post-operatively							
No side effects (*n* = 77)	15 (55.5%)	22 (75.8%)	40 (91%)	0.003	0.121	0.001	0.072
Dizziness (*n* = 5)	3 (11.1%)	0 (0%)	2 (4.5%)				
Headache (*n* = 11)	7 (25.9%)	3 (10.3%)	1 (2.3%)				
Sweating (*n* = 2)	1 (3.7%)	1 (3.4%)	0 (0%)				
Dry Mouth (*n* = 5)	1 (3.7%)	3 (10.3%)	1 (2.3%)				
At 24 h post-operatively							
No side effects (*n* = 77)	14 (51.8%)	24 (88.8%)	39 (88.6%)	0.003	0.019	0.002	0.310
Dizziness (*n* = 5)	3 (11.1%)	0 (0%)	2 (4.5%)				
Headache (*n* = 12)	8 (29.6%)	2 (6.8%)	2 (4.5%)				
Sweating (*n* = 3)	1 (3.7%)	2 (6.8%)	0 (0%)				
Dry Mouth (*n* = 3)	1 (3.7%)	1 (3.4%)	1 (2.3%)				

## Data Availability

All the relevant data have been provided in the manuscript. Data used and/or analyzed during the current study are available from the corresponding author upon reasonable request.
